# Differential Inhibition of *Ex-Vivo* Tumor Kinase Activity by Vemurafenib in *BRAF*(V600E) and *BRAF* Wild-Type Metastatic Malignant Melanoma

**DOI:** 10.1371/journal.pone.0072692

**Published:** 2013-08-30

**Authors:** Andliena Tahiri, Kathrine Røe, Anne H. Ree, Rik de Wijn, Karianne Risberg, Christian Busch, Per E. Lønning, Vessela Kristensen, Jürgen Geisler

**Affiliations:** 1 Department of Clinical Molecular Biology and Laboratory Sciences, Akershus University Hospital, Lørenskog, Norway; 2 Institute of Clinical Medicine, University of Oslo, Oslo, Norway; 3 Department of Oncology, Akershus University Hospital, Lørenskog, Norway; 4 PamGene International B.V., ‘s- Hertogenbosch, The Netherlands; 5 Section of Oncology, Institute of Medicine, University of Bergen, Bergen, Norway; 6 Department of Oncology, Haukeland University Hospital, Bergen, Norway; IDI, Istituto Dermopatico dell’Immacolata, Italy

## Abstract

**Background:**

Treatment of metastatic malignant melanoma patients harboring *BRAF*(V600E) has improved drastically after the discovery of the *BRAF* inhibitor, vemurafenib. However, drug resistance is a recurring problem, and prognoses are still very bad for patients harboring *BRAF* wild-type. Better markers for targeted therapy are therefore urgently needed.

**Methodology:**

In this study, we assessed the individual kinase activity profiles in 26 tumor samples obtained from patients with metastatic malignant melanoma using peptide arrays with 144 kinase substrates. In addition, we studied the overall *ex-vivo* inhibitory effects of vemurafenib and sunitinib on kinase activity status.

**Results:**

Overall kinase activity was significantly higher in lysates from melanoma tumors compared to normal skin tissue. Furthermore, *ex-vivo* incubation with both vemurafenib and sunitinib caused significant decrease in phosphorylation of kinase substrates, i.e kinase activity. While basal phosphorylation profiles were similar in *BRAF* wild-type and *BRAF*(V600E) tumors, analysis with *ex-vivo* vemurafenib treatment identified a subset of 40 kinase substrates showing stronger inhibition in *BRAF*(V600E) tumor lysates, distinguishing the *BRAF* wild-type and *BRAF*(V600E) tumors. Interestingly, a few *BRAF* wild-type tumors showed inhibition profiles similar to *BRAF*(V600E) tumors. The kinase inhibitory effect of vemurafenib was subsequently analyzed in cell lines harboring different *BRAF* mutational status with various vemurafenib sensitivity *in-vitro*.

**Conclusions:**

Our findings suggest that multiplex kinase substrate array analysis give valuable information about overall tumor kinase activity. Furthermore, intra-assay exposure to kinase inhibiting drugs may provide a useful tool to study mechanisms of resistance, as well as to identify predictive markers.

## Introduction

Metastatic malignant melanoma is associated with a poor prognosis. For decades, patients have been treated with palliative chemotherapy like dacarbazine (DTIC) monotherapy. However, only 10–15% of patients respond to this type of treatment, and for the majority, responses are of short duration only [1].

Recent advances in melanoma research have unraveled some of the complexity of the molecular mechanisms of this disease. The oncogene *BRAF* (v-raf murine sarcoma viral oncogene homolog B1) is frequently mutated in melanoma (40–50% of cases) and has resulted in the development of *BRAF*-targeting kinase inhibitors, like vemurafenib (PLX4032) and dabrafenib [2]–[4]. Following the recent approvals by both the Food and Drug Administration and the European Medicines Agency, vemurafenib is now increasingly used for treatment of patients with late-stage melanoma harboring *BRAF*(V600E/K) mutations. Initial clinical studies with vemurafenib showed remarkably positive results, with response rates approaching 80% [2]. However, the duration of response was recognized to last for a few months only [2], and occurrence of drug resistance was a major drawback [5], [6]. Thus, better understanding of the molecular mechanisms involved in resistance to vemurafenib therapy may identify interacting tumor signaling pathways that further can be exploited as alternative actionable therapy targets.

Kinases have become attractive targets for novel anticancer drugs [7]–[10]. Tumor kinase signaling comprising composite activities of effector proteins, both directly down-stream in the *BRAF*-signaling pathway, but also more indirectly participating within this particular signaling pathway, might be implicated in cancer progression and drug resistance, and could act as functional biomarkers. In this study, we assayed the kinase activity in protein lysates from tumor samples provided by metastatic melanoma patients using a multiplex kinase substrate array technology. Our primary aim was to identify specific kinase activity profiles of metastatic melanoma and normal skin tissue. Secondly, we aimed to study the *ex-vivo* inhibitory effects of vemurafenib in order to describe kinases and signaling pathways involved in vemurafenib response, and to compare the findings to the inhibitory effects of *in-vitro* vemurafenib treatment in metastatic melanoma cell lines. The experiments were repeated with sunitinib, a multi-targeted kinase inhibitor, for comparison of results obtained with vemurafenib, using the same methodological approach.

## Materials and Methods

### Ethics Statement

The Regional Committee for Medical and Health Research Ethics approved the study, and each patient provided written informed consent.

### Tissue Specimens

In total, 26 fresh-frozen tumor samples from patients suffering from stage IV melanoma were collected prior to DTIC treatment at Haukeland University Hospital (Table 1). The patient material was collected from October 1999 to November 2007, and follow-up was terminated in May 2009. The tumor biopsies were collected from distant metastases or from locoregional relapse by incisional or tru-cut (liver) biopsies, and were immediately snap-frozen in liquid nitrogen (individual patient characteristics are summarized in Table S1). All tissue specimens have been histologically confirmed by a pathologist and have previously been described and screened for mutations in *BRAF*, *NRAS* (neuroblastoma RAS viral (v-ras) oncogene homolog), *CDKN2A* (cyclin-dependent kinase inhibitor 2A), and *TP53* (Tumor protein p53) [11]–[13]. Additionally, four normal skin tissue samples were collected at Akershus University Hospital in 2010 from individuals not affected by melanoma. No clinical data was obtained from these patients.

**Table 1:** Patient Characteristics.

	All patients (n)	*BRAF* wild-type (n)	*BRAF*(V600E) (n)
**Patient demographic**			
Number of samples	26	15	10
Age at diagnosis, median (yrs)	60	59	61
Age at metastasis, median (yrs)	65	65	65
**Sex**			
Male	14	7	4
Female	11	8	6
**Localization of primary tumor**			
Lower extremity	6	4	2
Upper extremity	3	3	-
Head	3	3	-
Trunk	9	2	7
No primary detected	4	3	1
**Pathological types of melanoma**			
Nodular melanoma	8	7	1
Superficial spreading	11	5	6
Unknown	7	4	3
**Type of metastasis**			
Lymph node	6	-	6
Subcutaneous	18	14	4
**Clinical stage at inclusion**			
Stage III	1	-	1
Stage IV	25	15	9
**Response to DTIC**			
Responder	13	9	4
Non-responder	13	7	6
***NRAS*** ** status**			
*NRAS* wild-type	20	10	10
*NRAS*(Q61)	6	6	-

### Tissue Preparation

The tissue specimens were sectioned with a microtome into 10 µm thick coupes, to a total volume of ∼3 mm^3^. The number of coupes needed was calculated based on the surface area of the tissue specimen. The tissue samples were kept frozen at all times during the procedure, and stored at −80°C until further use. To avoid contamination, the tumor and normal tissue specimens were prepared separately. The sectioned tissue was lysed with the mammalian protein extraction reagent (M-PER) buffer (Pierce Biotechnology, Inc., Rockford, IL), supplemented with phosphatase and protease inhibitors (Pierce Biotechnology, Inc), for the determination of kinase activity profiles in the presence and absence of two different inhibitors; vemurafenib (PLX4032; Axon Medchem B.V., Groningen, The Netherlands) and sunitinib (SU11248; Sigma Aldrich, Oslo, Norway). The protein concentration of lysates was determined using the BCA assay (Pierce Biotechnology, Inc.). For each experiment, 15 µg of protein lysate from melanoma tissue or 20 µg of protein lysate from normal skin tissue was added to the reaction mixture, in addition to 400 µM ATP and 12.5 mg/mL of monoclonal fluorescein isothiocyanate-conjugated anti- phosphotyrosine antibody (Exalpha Biologicals, Inc., Maynard, MA).

### Kinase Activity Profiling of Metastatic Malignant Melanoma Tumors

Kinase activity profiling was performed using the Tyrosine Kinase PamChip® Array for Pamstation®12 (PamGene International B.V., ‘s-Hertogenbosch, The Netherlands) at Akershus University Hospital. Each array consists of 144 peptide substrates, primarily with known tyrosine residues, representing ∼100 different proteins. Three chips can be run simultaneously, and each chip consists of four arrays. The lysates are repeatedly pumped up and down through the porous array, allowing repeat substrate phosphorylation. Based on pilot experiments of increasing concentrations of the individual kinase inhibitors added to melanoma tissue lysates that were incubated on the arrays, concentrations that resulted in ∼50% inhibition of most kinase substrates were chosen for the main experiments. Hence, concentrations of 40 µM vemurafenib and 7.5 µM sunitinib were spiked into the assay mixtures prior to incubation, whereas 1.5% dimethyl sulfoxide was added to mixtures not containing the inhibitors. The samples were run in three technical replicates in the presence or absence of vemurafenib, as paired measurements with and without inhibitor on the same chip. The experimental procedure was repeated with sunitinib. Incubations were commenced for 60 cycles, followed by washing and fluorescence measurement of all peptide spots every fifth cycle. The experiments were run blinded, and the tumor and normal skin tissue lysates were run separately. The microarray data are submitted to ArrayExpress (http://www.ebi.ac.uk/arrayexpress/); accession number E-MTAB-1245.

### Data Adaptation and Statistical Analysis of Malignant Melanoma Tumors

End-level signal intensities for each peptide spot were quantified and analyzed using BioNavigator version 5.1 (Pamgene International B.V.). Signals obtained after subtraction of local array background were used for further analysis. Negative numbers were set to 0.01 and log_2_-transformed.

For analysis of the ‘basal kinase activity profiles’ (measurements obtained without inhibitor), the technical replicates were averaged. The overall difference between the measurements in the first series, relative to that in the second series was corrected by subtracting the mean of each peptide in the corresponding experimental series (centering), and by averaging the centered results of both experimental series. For analysis of ‘inhibition profiles’ (measurement obtained with inhibitor), values were obtained by calculating the log-fold change (LFC) of each peptide without any further normalization of the data. LFC was calculated by subtracting the log_2_-transformed signal values with inhibitor from the corresponding values without inhibitor added. The pairing of measurements with and without inhibitor was taken into account by first calculating the LFC of each chip, and subsequently averaging the LFCs of each chip to obtain the value used in further analysis.

Per-peptide differences between conditions were evaluated using two-tailed *t*-tests, and unsupervised multivariate clustering of samples was evaluated with principal component analysis (PCA), both using BioNavigator interfaced to R (The R-project). Supervised multivariate analysis of conditions was performed by applying partial least squares discriminant analysis (PLS-DA), using BioNavigator interfaced to a custom PLS-DA implementation written in Matlab (MathWorks, Natick, MA). PLS-DA was performed without any pre-selection of kinase substrates. Prediction performance was evaluated using leave-one-out cross-validation (LOOCV), making sure that the model was optimized completely independent of the test sample [14]. Pathway connectivity of kinase substrates was determined by using the KEGG pathway database [15], [16] and literature search.

### Kinase Activity Profiling and Statistical Analysis of Melanoma Cell Lines

The MelJD, patient-3-post and MM200 metastatic melanoma cell lines were obtained from Professor P. Hersey, University of Sydney, Sydney, NSW, Australia [17], [18]. The MelJD cell line is *BRAF* wild-type, whereas the patient-3-post and MM200 cell lines harbor the *BRAF*(V600E) mutation. In this manuscript we entitle the patient-3-post cell line as “vemurafenib-resistant” and the MM200 cell line as “vemurafenib-sensitive”, due to their difference in sensitivity to vemurafenib treatment, as also shown previously [17], [18]. All cell lines were maintained in RPMI 1640 medium (Sigma-Aldrich, Oslo, Norway) supplemented with 10% fetal calf serum and 1% Glutamax (Invitrogen, Oslo, Norway). The cells were routinely grown as a monolayer in 75 cm^2^ flasks at 37°C in 95% air/5% CO_2_, and subcultured twice a week to maintain exponential growth. The cell lines were confirmed to be mycoplasma-free prior to the experiments.

Cells were exposed to *in-vitro* treatment with vemurafenib (5 µM) or dimethyl sulfoxide (vehicle) for 1 hour. The cells were harvested by washing the cells twice with 10 ml ice-cold PBS, before adding 4 ml ice-cold PBS and loosening the cells by scraping. To obtain the pellet, the samples were centrifuged (10 minutes, 2500 rpm, 4°C) and supernatant was removed. Lysis buffer was added and the samples were vortexed and lysed for 15 minutes on ice. After centrifugation (15 minutes, 15000 rpm, 4°C), supernatants were aliqouted and immediately frozen at −80°C. Protein concentrations were measured using a BCA protein assay kit (Pierce Biotechnology, Inc).

Kinase activity profiling was assessed by using 10 µg of total protein from all samples. Lysates from each cell line were run in triplicates. The raw data was log_2_-transformed by identical procedures as the data from the patient specimens, before per-peptide differences between conditions (vemurafenib-treated versus untreated samples, and pair-wise comparison of cell lines) were evaluated using the two-tailed *t*-tests.

## Results

### Basal Kinase Activity in Metastatic Malignant Melanoma

**Figure 1:**
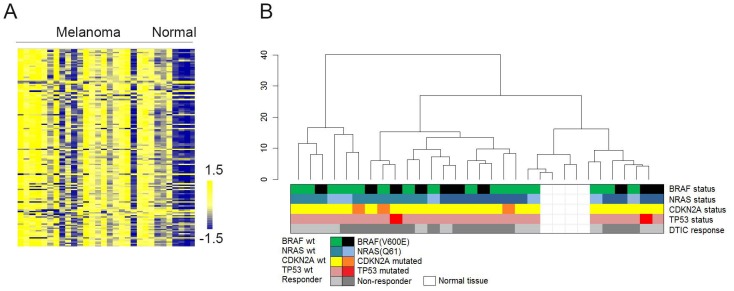
Kinase activity profiles of metastatic malignant melanoma and normal skin tissue. **A)** The heat map shows phosphorylation levels for all 144 kinase substrates (vertical axis) in response to incubation with lysates from metastatic malignant melanoma samples and normal skin tissue samples (horizontal axis). Color bar represents phosphorylation intensities; blue indicates low phosphorylation levels, whereas yellow indicates higher phosphorylation levels. **B)** Unsupervised hierarchical clustering including all samples and 144 kinase substrates did not reveal any correlation between phosphorylation profiles and different molecular and clinical parameters. Different variables are indicated by colors, including *BRAF*-, *NRAS*-, *CDKN2A*-, *TP53*- mutational status, and DTIC response.

The majority of the array kinase substrates (80–90%) showed higher phosphorylation levels upon incubation with metastatic melanoma lysates compared with normal skin tissue lysates. The difference ranged up to 5-fold between the two tissue types (Figure 1A and Table S2). Supervised and unsupervised clustering analysis of the samples showed no correlations between phosphorylation patterns of kinase substrates and known molecular (*BRAF*-, *NRAS*-, *CDKN2A*-, or *TP53* mutational status) or clinical parameters (age, gender, stage, or anatomical location of tumor), including response to DTIC (Figure 1B).

### *Ex-vivo* Kinase Inhibitory Effects of Vemurafenib

The inhibition profiles obtained with *ex-vivo* exposure of melanoma tumor lysates to vemurafenib showed reduced kinase substrate phosphorylation levels. Whilst phosphorylation levels of the majority of the kinase substrates were decreased by approximately 50% (Table S3), the inhibitory effect was weaker on kinase substrates with low basal phosphorylation levels.

**Figure 2:**
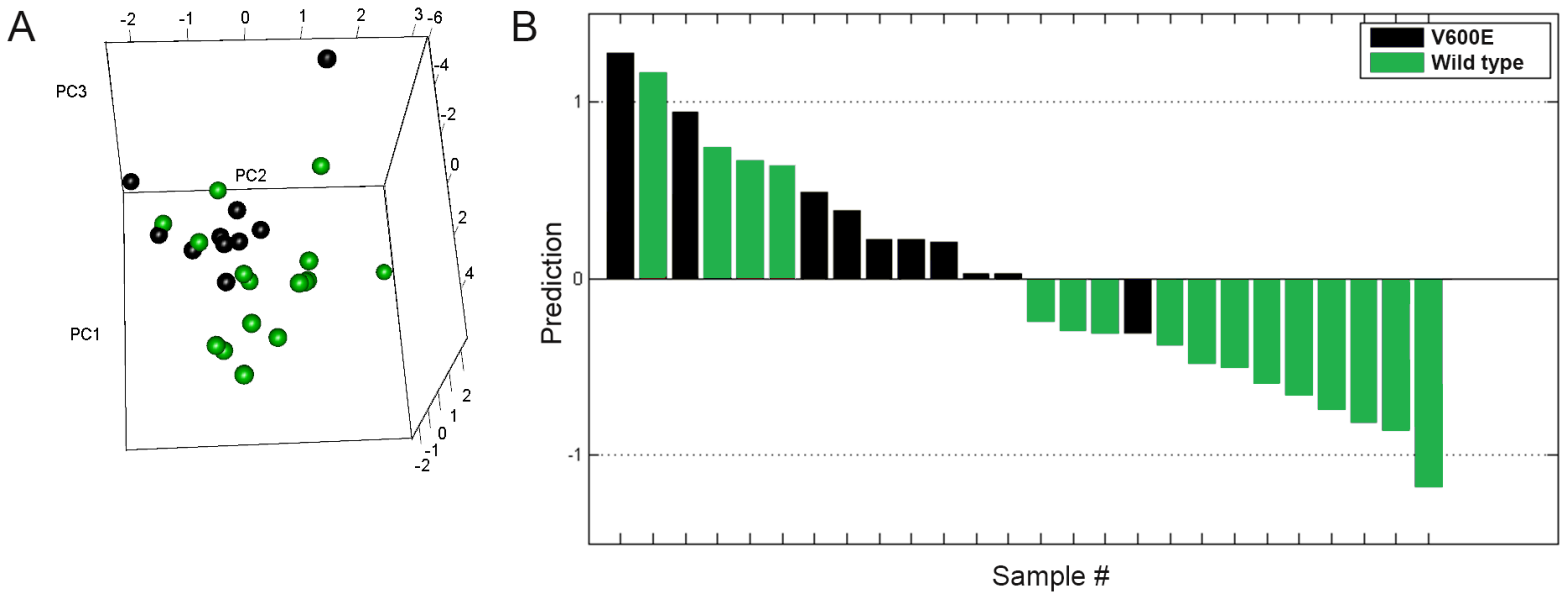
Classification of melanoma samples based on *BRAF* mutational status. **A)** Unsupervised principal component analysis (PC1–3) including all 144 kinase substrates separated *BRAF* wild-type (green) and *BRAF*(V600E) (black) melanoma tumors in two groups based on the inhibition profiles obtained with *ex-vivo* vemurafenib. **B)** *BRAF* wild-type (green) and *BRAF*(V600E) (black) melanoma tumors were classified with partial least squares discriminant analysis. The prediction scores shown were obtained by testing the corresponding sample during leave-one-out cross-validation. Samples with prediction score lower than 0 were classified as *BRAF wild-type*, whereas samples with prediction score higher than 0 were classified as *BRAF*(V600E).

*BRAF*(V600E) mutation was present in 10 out of 26 tumors (38.5%), whereas *NRAS*(Q61) mutation was present in 6 out of 26 tumors (23%). Unsupervised PCA showed a tendency of separation between *BRAF*(V600E) and *BRAF* wild-type tumors (Figure 2A). Prediction performance with PLS-DA was evaluated using LOOCV [14]. This type of supervised analysis classified *BRAF* wild-type and *BRAF*(V600E) samples based on the inhibition profiles, with an accuracy of 20/26 samples (77%) (Figure 2B). Classification of tumors harboring *BRAF*(V600E) was correct for 90% of the samples, whereas for *BRAF* wild-type tumors, only 75% of samples were correctly classified with PLS-DA, reflecting the interesting observation that a few *BRAF* wild-type tumors consistently grouped with *BRAF*(V600E) tumors.

**Figure 3:**
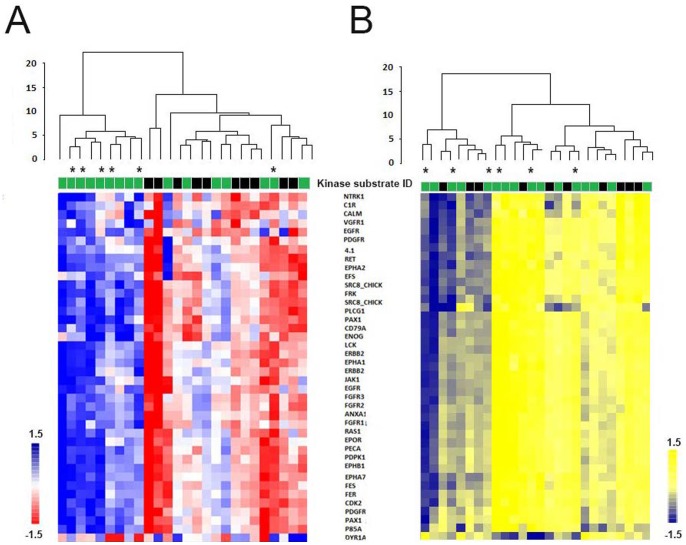
Supervised clustering of *BRAF*(V600E) and *BRAF* wild-type melanoma tumors. **A)** Supervised clustering of melanoma samples based on 40 kinase substrates (vertical axis) identified as significantly differentially affected by *ex-vivo* exposure to vemurafenib in *BRAF* wild-type (green) and *BRAF*(V600E) (black) samples (horizontal axis). Clustering using the inhibition profiles separated the samples in two groups according to *BRAF* mutational status. Color bar represents the level of inhibition; red indicates strong inhibition, whereas blue indicates weak inhibition. **B)** Clustering using the basal kinase activity data did not separate the melanoma samples according to *BRAF* mutational status. Color bar represents the level of phosphorylation; yellow indicates high phosphorylation, whereas blue indicates low phosphorylation of kinase substrates. Samples marked with asterisks (*) harbor *NRAS* mutations.

**Figure 4:**
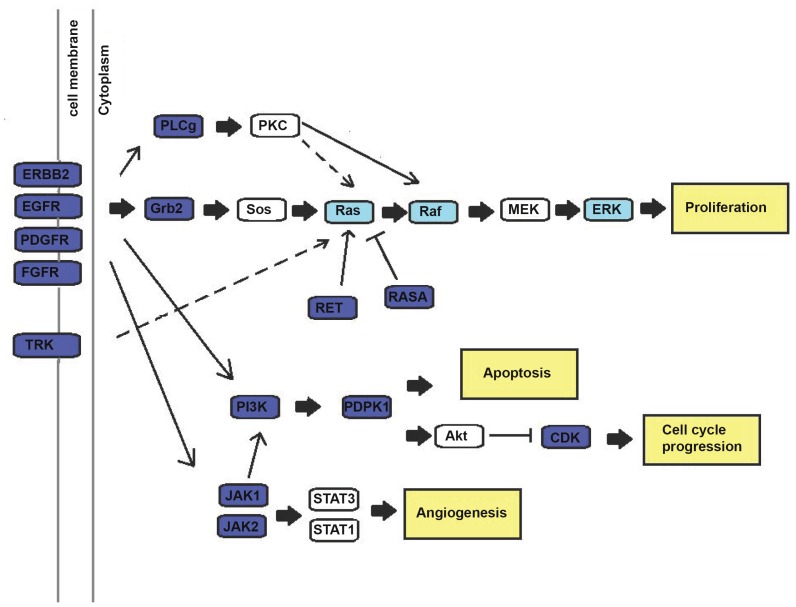
Kinases and pathways affected by *ex-vivo* vemurafenib in *BRAF*(V600E) melanoma tumors. In dark blue color are array substrates representing kinases distinguishing between *BRAF* wild-type and *BRAF*(V600E) tumors (*P*<0.05) in response to vemurafenib. Marked with light blue color are kinase substrates showing reduced levels of phosphorylation in response to vemurafenib, but which are not identified as differentially inhibited according to *BRAF* mutational status. In yellow color are the main cellular processes (angiogenesis, apoptosis, proliferation, and cell cycle progression) affected in response to *ex-vivo* vemurafenib. Some kinase substrates may be represented in more than one cellular process. Note that RAF in this case is CRAF, not BRAF. **Abbreviations**: v-akt murine thymoma viral oncogene (AKT), cyclin-dependent kinase (CDK), epidermal growth factor receptor (EGFR), v-erb-b2 erythroblastic leukemia viral oncogene homolog 2 (ERBB2), extracellular-signal-regulated kinases (ERK), fibroblast growth factor receptor (FGFR), growth factor receptor-bound protein 2 (Grb2), janus kinase (JAK), mitogen-activated protein kinase kinase (MEK), platelet-derived growth factor receptor (PDGFR), 3-phosphoinositide dependent protein kinase-1(PDK1), phosphatidylinositide 3-kinase (PI3K), protein kinase C (PKC), phospholipase C- gamma (PLCg), v-Raf murine sarcoma viral oncogene (RAF), rat sarcoma viral oncogene (RAS), ret proto-oncogene (RET), son of sevenless (SOS), signal transducer and activator of transcription (STAT), neurotrophic tyrosine kinase receptor (TRK).

Furthermore, applying two-tailed *t*-tests identified 40 kinase substrates that were significantly affected by *ex-vivo* vemurafenib (*P*<0.05), and distinguished between *BRAF*(V600E) and *BRAF* wild-type tumors (Table S4). Supervised clustering analysis comprising these 40 kinase substrates showed a significantly stronger inhibitory effect of vemurafenib in *BRAF*(V600E) tumors than in *BRAF* wild-type tumors (Figure 3A). Again, a few *BRAF* wild-type tumors invariably grouped together with *BRAF*(V600E) tumors, exhibiting stronger inhibition in response to vemurafenib than the other *BRAF* wild-type tumors. No statistically significant differences in phosphorylation profiles were observed between *BRAF*(V600E) and *BRAF* wild-type tumors in the absence of *ex-vivo* vemurafenib incubation (Figure 3B). The kinase substrates that distinguished between *BRAF* wild-type and *BRAF*(V600E) tumors represented kinases mainly involved in the phosphatidylinositide 3-kinase (PI3K) and mitogen-activated protein kinase (MAPK) signaling network, including processes such as angiogenesis, proliferation, cell cycle progression and apoptosis (Figure 4 and Table S5). However, pathway exploration including all 144 peptides revealed that both these pathways were overrepresented on the array (Table S5).

### *Ex-vivo* Kinase Inhibitory Effects of Sunitinib

**Figure 5:**
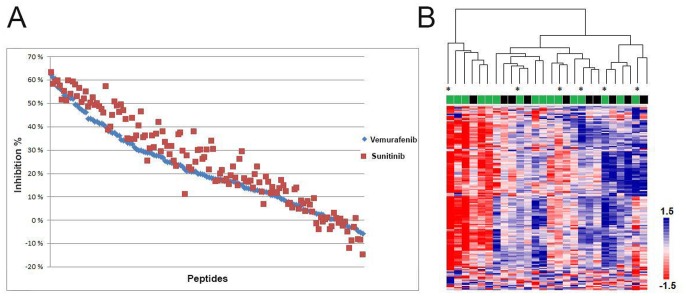
Kinase inhibition profiles in response to *ex-vivo* exposure to vemurafenib or sunitinib. **A)** Inhibition (y-axis) of all 144 kinase substrates (x-axis) in response to *ex-vivo* incubation with vemurafenib and sunitinib in metastatic malignant melanoma tumors. **B)** Heat map with sunitinib inhibition profiles of all 144 kinase substrates (vertical) and twenty-six metastatic malignant melanoma tumors (horizontal). Unsupervised hierarchical clustering did not show any correlation with *BRAF*- (*BRAF* wild-type (green), *BRAF*(V600E) (black)) or *NRAS*(Q61) (marked with *) mutations. Color bar represents inhibition intensities; red indicates strong inhibition, whereas blue indicates weak inhibition.

Sunitinib, a multi-targeted receptor tyrosine kinase inhibitor, affected as expected a range of array kinase substrates, revealing inhibition profiles resembling those obtained with vemurafenib (Figure 5A and Table S3). However, in contrast to the results obtained with vemurafenib inhibition, attempts to correlate sunitinib inhibition profiles to *BRAF* mutational status or other molecular or clinical parameters, including supervised analysis with the panel of 40 kinase substrates, showed no significant findings. Unsupervised hierarchical clustering with inhibition profiles including all samples and kinase substrates is shown in Figure 5B.

### *In-vitro* Inhibitory Effects of Vemurafenib on Kinase Activity in *BRAF*(V600E) and *BRAF* Wild-type Melanoma Cell Lines

To further examine the role of *BRAF* mutational status on kinase activity, and as a mean to validate results obtained with patient specimens, we profiled the kinase activity of lysates from the three melanoma cell lines MelJD (*BRAF* wild-type), patient-3-post (*BRAF*(V600E)/“vemurafenib-resistant”) and MM200 (*BRAF*(V600E)/“vemurafenib-sensitive”).

**Figure 6:**
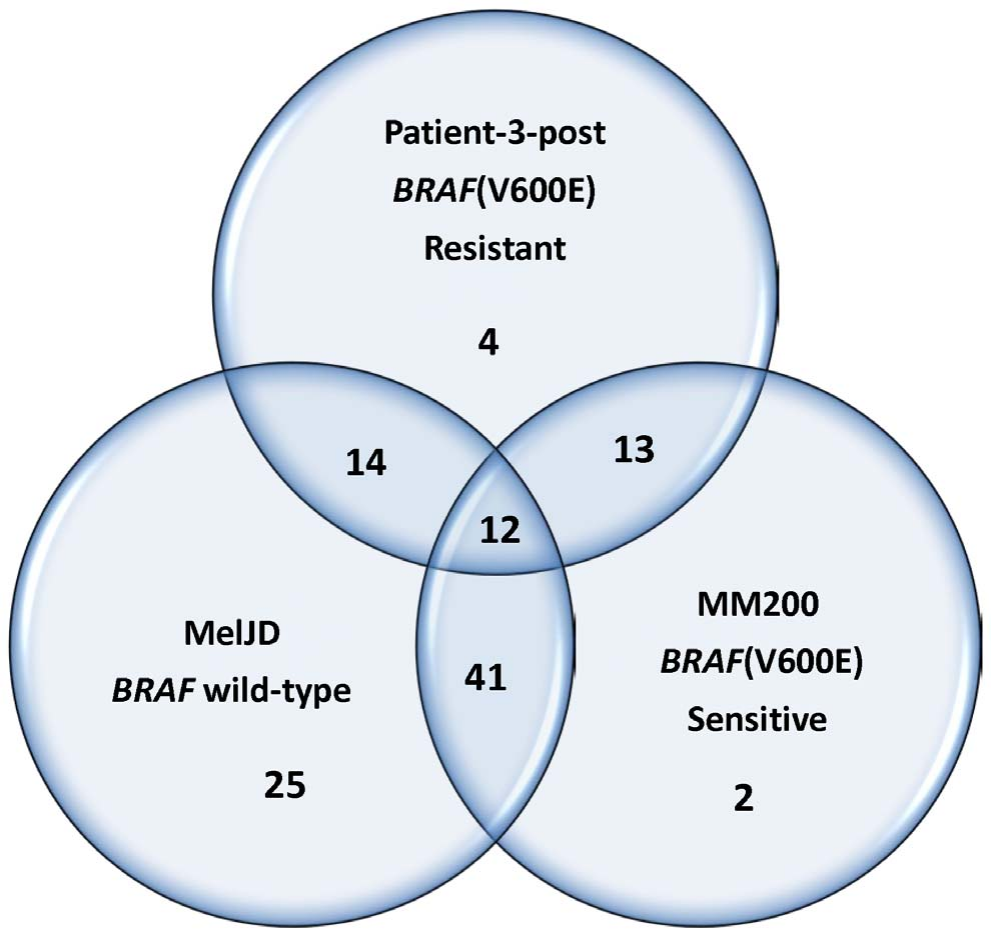
Venn diagram of kinase substrates that are significantly affected by vemurafenib in BRAF(V600E) and BRAF wild-type melanoma cell lines. The MelJD cells harbor BRAF wild-type, whereas both patient-3-post and MM200 cells harbor BRAF(V600E) mutations. The patient-3-post cells are vemurafenib-resistant, whereas MM200 cells are sensitive to vemurafenib. The numbers given denote the number of kinase substrates that are significantly affected in each pair-wise comparison of the three different cell lines, as well as the number of kinase substrates that are commonly affected among the cell lines.

The results showed a great variability in the reduction of kinase substrate phosphorylation levels in response to *in-vitro* vemurafenib treatment. Reduced phosphorylation levels were seen in all cell lysates, with the largest effect seen in lysates from the *BRAF* wild-type MelJD cells (Table S6). Inhibition profiles revealed that the phosphorylation levels of only 12 kinase substrates were significantly (*P*<0.05) reduced by vemurafenib in all three cell lines (Figure 6). Stronger kinase inhibition after vemurafenib treatment was seen in MelJD and MM200 cells, with 41 of the same kinase substrates affected. MelJD and vemurafenib-sensitive MM200 cells showed similar inhibition profiles compared to vemurafenib-resistant cells. In total, the phosphorylation level of 59 kinase substrates were significantly differentially affected by vemurafenib (*P*<0.05) in patient-3-post and MelJD cells, with 50% (20/40) of them being identical to the kinase substrates identified as differentially affected between *BRAF* wild-type and *BRAF*(V600E) in patient specimens (Table 2). A smaller number of kinase substrates were differentially affected by vemurafenib in MM200 and MelJD cells, respectively, with only 20% (8/40) of the kinase substrates being the same as the ones identified in patient specimens.

**Table 2:** Significantly differentially affected kinase substrates (*P*<0.05) between *BRAF*(V600E) and *BRAF* wild-type melanoma in lysates from cell lines and tumor tissue.

Kinase substrate ID	Encoding protein	MelJD vs MM200	MelJD vs patient-3-post	Melanoma tissue
41_654_666	Erythrocyte membrane protein band 4.1		**X**	X
ANXA1_14_26	Annexin A1	**X**	**X**	X
ANXA2_17_29	Annexin A2 pseudogene 3; annexin A2; annexin A2 pseudogene 1		X	
C1R_199_211	Complement component 1, r subcomponent		**X**	X
CALM_93_105	Calmodulin 3; calmodulin 2; calmodulin 1			X
CD3Z_116_128	CD247 molecule		X	
CD79A_181_193	CD79a molecule, immunoglobulin-associated alpha	**X**	**X**	X
CDK2_8_20	Cyclin-dependent kinase 2			X
CDK7_157_169	Cyclin-dependent kinase 7		X	
CRK_214_226	v-crk sarcoma virus CT10 oncogene homolog	X	X	
CTNB1_79_91	Catenin (cadherin-associated protein), beta		X	
DCX_109_121	Doublecortin		X	
DYR1A_312_324	Dual-specificity tyrosine-(Y)-phosphorylation regulated kinase 1A	**X**	**X**	X
EFS_246_258	Embryonal Fyn-associated substrate			X
EGFR_1062_1074	Epidermal growth factor receptor			X
EGFR_1103_1115	Epidermal growth factor receptor	X	X	
EGFR_1165_1177	Epidermal growth factor receptor		X	
EGFR_1190_1202	Epidermal growth factor receptor	X	X	
EGFR_862_874	Epidermal growth factor receptor	**X**		X
ENOG_37_49	Enolase 2 (gamma, neuronal)			X
EPHA1_774_786	EPH receptor A1	**X**	**X**	X
EPHA2_581_593	EPH receptor A2		X	
EPHA2_765_777	EPH receptor A2			X
EPHA7_607_619	EPH receptor A7		**X**	X
EPHB1_771_783	EPH receptor B1		**X**	X
EPOR_361_373	Erythropoietin receptor			X
EPOR_419_431	Erythropoietin receptor		X	
ERBB2_1241_1253	v-erb-b2 erythroblastic leukemia viral oncogene homolog 2			X
ERBB2_870_882	v-erb-b2 erythroblastic leukemia viral oncogene homolog 2			X
FAK1_569_581	PTK2 protein tyrosine kinase 2	X	X	
FAK2_572_584	PTK2B protein tyrosine kinase 2 beta	X	X	
FER_707_719	Fer (fps/fes related) tyrosine kinase		**X**	X
FES_706_718	Feline sarcoma oncogene			X
FGFR1_761_773	Fibroblast growth factor receptor 1			X
FGFR2_762_774	Fibroblast growth factor receptor 2			X
FGFR3_753_765	Fibroblast growth factor receptor 3	**X**	**X**	X
FRK_380_392	Fyn-related kinase	**X**	**X**	X
JAK1_1015_1027	Janus kinase 1		**X**	X
JAK2_563_577	Janus kinase 2		X	
K2C6B_53_65	Keratin 6B	X	X	
K2C8_425_437	Keratin 8 pseudogene 9	X	X	
LAT_194_206	Linker for activation of T cells	X	X	
LAT_249_261	Linker for activation of T cells	X	X	
LCK_387_399	Lymphocyte-specific protein tyrosine kinase	**X**		X
MET_1227_1239	Met proto-oncogene (hepatocyte growth factor receptor)	X	X	
MK01_180_192	Mitogen-activated protein kinase 1	X	X	
MK07_211_223	Mitogen-activated protein kinase 7	X	X	
MK14_173_185	Mitogen-activated protein kinase 14	X	X	
NPT2A_501_513	Solute carrier family 34 (sodium phosphate), member 1	X	X	
NTRK1_489_501	Neurotrophic tyrosine kinase, receptor, type 1			X
NTRK2_696_708	Neurotrophic tyrosine kinase, receptor, type 2	X	X	
P85A_600_612	Phosphoinositide-3-kinase, regulatory subunit 1 (alpha)		**X**	X
PAXI_111_123	Paxillin		**X**	X
PAXI_24_36	Paxillin			X
PDPK1_2_14	3-phosphoinositide dependent protein kinase-1			X
PDPK1_369_381	3-phosphoinositide dependent protein kinase-1	X	X	
PECA1_706_718	Platelet/endothelial cell adhesion molecule			X
PGFRB_1002_1014	Platelet-derived growth factor receptor, beta polypeptide		X	
PGFRB_1014_1028	Platelet-derived growth factor receptor, beta polypeptide		**X**	X
PGFRB_572_584	Platelet-derived growth factor receptor, beta polypeptide			X
PGFRB_768_780	Platelet-derived growth factor receptor, beta polypeptide		X	
PGFRB_771_783	Platelet-derived growth factor receptor, beta polypeptide		X	
PLCG1_764_776	Phospholipase C, gamma 1		**X**	X
PRRX2_202_214	Paired related homeoboX 2	X	X	
RAF1_332_344	v-raf-1 murine leukemia viral oncogene homolog 1	X		
RASA1_453_465	RAS p21 protein activator (GTPase activating protein) 1			X
RET_1022_1034	Ret proto-oncogene		**X**	X
RON_1346_1358	Macrophage stimulating 1 receptor (c-met-related tyrosine kinase)		X	
SRC8_CHICK_476_488	Cortactin		**X**	X
SRC8_CHICK_492_504	Cortactin		**X**	X
STAT1_694_706	Signal transducer and activator of transcription 1		X	
STAT4_714_726	Signal transducer and activator of transcription 4		X	
TEC_512_524	Tec protein tyrosine kinase		X	
TYRO3_679_691	TYRO3 protein tyrosine kinase	X	X	
VGFR1_1040_1052	Fms-related tyrosine kinase 1 (vascular endothelial growth factor)		X	
VGFR1_1049_1061	Fms-related tyrosine kinase 1 (vascular endothelial growth factor)	X	X	
VGFR1_1235_1247	Fms-related tyrosine kinase 1 (vascular endothelial growth factor)		**X**	X
VGFR2_1046_1058	Kinase insert domain receptor (a type III receptor tyrosine kinase)	X		
VGFR2_1052_1064	Kinase insert domain receptor (a type III receptor tyrosine kinase)		X	
ZAP70_485_497	Zeta-chain (TCR) associated protein kinase	X	X	

X denotes the kinase substrates that are significantly affected between *BRAF*(V600E) and *BRAF* wild-type.

X highlighted in bold denotes kinase substrates that were also identified as significant in melanoma tissue.

## Discussion

Following several decades of nearly complete stagnation in the clinical treatment of patients with metastatic malignant melanoma, we are currently witnessing dramatic improvements regarding therapy. Activating mutations in *BRAF* (mainly V600E/K) have been identified in half of all melanoma cases [4], and the development of novel compounds targeting mutated *BRAF* [6], [19], or compounds boosting the immunological responses directed towards cancer cells [20], has given new hope to this group of patients. However, these studies have also revealed that early drug resistance occurs in the majority of patients, causing a considerable clinical challenge [2], [5], [6]. As kinases are part of key cellular process, and mutations herein are often implicated in both cancer progression and/or drug resistance [21], we examined the overall kinase activity profiles in metastatic malignant melanoma tumor samples, using a multiplex microarray technology previously proven to be robust and reliable [22]–[25].

We show that phosphorylation levels of kinase substrates were generally increased in lysates from metastatic melanoma compared to normal skin tissue, indicating high kinase activity. The phosphorylation patterns in melanoma did however not correlate to any clinical or molecular parameters, like *BRAF*- and *NRAS* mutational status, or response to DTIC therapy. The increased kinase activity observed in our melanoma samples is in agreement with previous studies which shows that kinases are hyperactive in many cancers, acting as a driving force towards tumor proliferation and other growth processes [7].

We further analyzed the *ex-vivo* inhibitory effects of the *BRAF* inhibitor, vemurafenib, and the multi-targeted tyrosine kinase inhibitor, sunitinib, in the same set of melanoma samples. Inhibition experiments with sunitinib were performed as an indication that the method worked as expected i.e. multiple kinase substrates were inhibited. Hence, no correlation in inhibition pattern was observed with regard to various clinical and biological parameters.

The *ex-vivo* inhibition profiles upon exposure to vemurafenib showed that a wide range of kinase substrates were affected, indicated by decreased levels of phosphorylation. This was also observed in cell lines treated with vemurafenib *in-vitro*, regardless of *BRAF* mutational status. Possible explanations for this comprehensive inhibitory pattern might be due to off-target effects of vemurafenib, as has been suggested by others [26]. Nevertheless, the majority of the affected substrates represented effector proteins participating within the signaling network mediating kinase activity through the *BRAF*-encoded pathway.

Interestingly, inhibition profiles obtained with *ex-vivo* vemurafenib revealed a panel of 40 kinase substrates distinguishing the *BRAF* wild-type and *BRAF*(V600E) melanoma tumors. Kinases involved in the PI3K and MAPK pathway were among the kinase substrates discriminating the two groups. However, bearing in mind that the 40 discriminating substrates in this analysis appeared from a total number of 144 peptides constituting the kinase activity profiles, the false discovery rate among peptides with a statistical significance level of *P*<0.05 can be estimated to be about 16%. The 40 kinase substrates were more strongly inhibited when incubated with lysates from tumors harboring *BRAF*(V600E) compared to *BRAF* wild-type tumors, which is consistent with previous studies showing that *BRAF*(V600E) tumors are more responsive to vemurafenib than *BRAF* wild-type tumors [4], [27], [28]. The unresponsiveness of *BRAF* wild-type tumors is thought to occur through a complex interplay between RAS and RAF dimers, leading to compensatory activation of the MAPK pathway [29]. Dimerization is promoted through RAS activation [30], and in the presence of activated NRAS, CRAF is preferred over BRAF, leading to loss of the inhibitory effect of vemurafenib [31]. Notably, *BRAF* and *NRAS* mutations are mutually exclusive in melanoma [32], which is also observed in our study. Thus, in *BRAF*(V600E) tumors, RAS activity is low, and the drug is able to bind to the BRAF monomer, blocking its activity completely.

In our study, both supervised and unsupervised analyses revealed that some wild-type *BRAF* samples also exhibited decreased levels of kinase substrate phosphorylation upon exposure to vemurafenib. These samples were classified together with the *BRAF*(V600E) tumors based on inhibition profiles. In the absence of clinical vemurafenib response data, we speculate whether these patients might benefit from vemurafenib treatment despite the lack of the V600E mutation. When profiling the kinase substrates in *BRAF* wild-type melanoma cell line (MelJD), we observed that kinase inhibition upon *in-vitro* vemurafenib treatment occurred to a similar degree as in the vemurafenib-sensitive cell line (MM200) harboring *BRAF*(V600E). This supports our findings from the patient specimens; that patients with wild-type *BRAF* may respond to vemurafenib treatment. Increased proliferation in *BRAF* wild-type cells in response to vemurafenib has, however, been reported as a possibly hazardous event [27], [29]. It would therefore be of interest to study these tumors for other activating mutations, either in the *BRAF* gene (e.g. *BRAF*(L597)) [33], or elsewhere, that has been shown to confer sensitivity to kinase inhibitors targeting the MAPK pathway.

Furthermore, some samples within the *BRAF*(V600E) group showed a lower degree of inhibition in *ex-vivo* response to vemurafenib. Lower degree of inhibition upon vemurafenib treatment *in-vitro* was also observed in our vemurafenib-resistant cell line (patient-3-post). This variability in sensitivity towards vemurafenib has previously been observed in several melanoma cell lines, where the presence of *BRAF* mutations did not guarantee a response [34], [35]. Further studies will be necessary to explore the potential of this differential degree of kinase inhibition in identifying patients that might respond poorly to vemurafenib, despite the presence of the indicative *BRAF*(V600E) mutation.

The 40 kinase substrate signature obtained between *BRAF* wild-type and *BRAF*(V600E) melanoma tumor samples after *ex-vivo* treatment with vemurafenib was similar to the signature obtained with *in-vitro* vemurafenib treatment between *BRAF* wild-type and vemurafenib-resistant *BRAF*(V600E) cells (Table 2). These results suggest that this signature may be useful in predicting patients benefiting from vemurafenib treatment.

Vemurafenib resistance is common in melanoma, and several mechanisms to how this occurs have been proposed. These include (a) *BRAF* splicing variants (p61*BRAF*(V600E)) lacking the RAS-binding domain [36]; (b) phosphatase and tensin homolog (*PTEN*) loss leading to elevated PI3K/AKT signaling [37]; (c) increased PDGFRβ expression leading to activation of survival pathways, or (d) *NRAS*(Q61K) mutations leading to activated MAPK pathway signaling [38]. In addition, increased EGFR expression in *BRAF*(V600E) colorectal tumors has been shown to correlate with vemurafenib resistance [39]. Expression of EGFR is generally low in melanoma compared to colorectal cancer [39]; however, EGFR overexpression in some melanoma tumors could explain the clinical resistance towards vemurafenib. Both EGFR and PDGFRβ are upstream of *BRAF* and affect both the MAPK and PI3K pathway (Figure 4). In our study, kinase substrates encoding for EGFR and PDGFRβ were found to be significantly differentially affected by *ex-vivo* vemurafenib in melanoma tumors harboring *BRAF*(V600E) and *BRAF* wild-type. This was also observed *in-vitro*, specifically in *BRAF* wild-type and vemurafenib-sensitive *BRAF(*V600E) cells, whereas no significant inhibition of EGFR and PDGFRβ was observed in the vemurafenib-resistant cell line (Table 3). Additionally, the kinase substrate encoding for RAF (C-RAF) was only significantly affected in the vemurafenib-sensitive cell line. Hence, our results support at least the notion that EGFR and PDGFRβ may be involved in the development of resistance to vemurafenib. Although resistance to BRAF inhibition is a challenge, recent evidence suggests that combinational therapy with inhibitors of the MAPK and PI3K pathway may be efficacious in melanoma patients with (V600E) mutations [5], [19], [40]. These findings make the signature of kinase substrates identified in this study as potential biomarker for such targeted therapy.

**Table 3:** EGFR, PDGFRβ and RAF kinase inhibitory effects of vemurafenib on lysates from cell lines harboring *BRAF*(V600E) mutations being resistant (patient-3-post) and sensitive (MM200) to vemurafenib, and *BRAF* wild-type (MelJD).

	*BRAF(*V600E) patient-3-post	*BRAF*(V600E) MM200	*BRAF* wild-type MelJD
Kinase substrate ID	*P* value	*P* value	*P* value
EGFR_1062_1074	3,71E-01	3,64E-01	1,84E-01
EGFR_1103_1115	2,10E-01	1,32E-01	**2,32E-02**
EGFR_1118_1130	1,14E-01	9,80E-01	6,97E-02
EGFR_1165_1177	6,90E-01	**2,06E-02**	**4,82E-02**
EGFR_1190_1202	5,20E-02	8,97E-01	**1,69E-02**
EGFR_862_874	3,99E-01	**1,01E-03**	4,49E-01
EGFR_908_920	8,03E-02	6,06E-01	2,96E-01
PGFRB_1002_1014	1,33E-01	**2,45E-02**	**1,21E-02**
PGFRB_1014_1028	1,72E-01	**2,15E-02**	**1,40E-03**
PGFRB_572_584	1,83E-01	8,84E-01	9,70E-02
PGFRB_709_721	**3,28E-02**	**1,51E-02**	**1,77E-02**
PGFRB_768_780	1,54E-01	**2,96E-02**	**2,18E-05**
PGFRB_771_783	2,12E-01	**2,84E-02**	**5,52E-03**
RAF1_332_344	8,41E-02	**2,46E-02**	5,11E-02

Highlighted in bold are kinase substrates with *P*<0.05.

In conclusion, our findings show that metastatic malignant melanoma is characterized by high activity of a range of kinases. The multiplex kinase substrate array technology used in the present study proved to be robust and reliable, and provided valuable information. This method may therefore become an important tool for screening of disease-specific functional biomarkers, and thereby pave the way for individualized cancer treatment. Furthermore, *ex-vivo* exposure to drugs may identify kinase substrate signatures that correlate to clinical response.

## Supporting Information

Table S1 Individual patient characteristics of all metastatic malignant melanoma cases.
(XLS)

Table S2 Mean phosphorylation intensity values of all kinase substrates across twenty-six metastatic malignant melanoma samples and four normal skin tissue samples in the basal data set.
(XLS)

Table S3 Mean inhibition of 144 kinase substrates in metastatic malignant melanoma samples in response to *ex-vivo* vemurafenib and sunitinib.
(XLS)

Table S4 Kinase substrates identified as significantly differentially affected (*P*<0.05) by ex-vivo vemurafenib incubation in *BRAF* wild-type and *BRAF*(V600E) melanoma samples.
(XLS)

Table S5 Results from KEGG pathway analysis of kinase substrates involved in the PI3K and MAPK signalling pathways including all 144 kinase substrates, and 40 kinase substrates differentiating between *BRAF*(V600E) and *BRAF* wild-type tumors.
(XLS)

Table S6 Kinase inhibitory effects of vemurafenib in melanoma cell lines harboring *BRAF*(V600E) mutations (MM200 and patient-3-post ) and *BRAF* wild type (MelJD).
(XLSX)

## References

[1] Serrone L, Zeuli M, Sega FM, Cognetti F (2000) Dacarbazine-based chemotherapy for metastatic melanoma: thirty-year experience overview. J Exp Clin Cancer Res 19: 21–34. 10840932

[2] Flaherty KT, Puzanov I, Kim KB, Ribas A, McArthur GA, et al (2010) Inhibition of mutated, activated BRAF in metastatic melanoma. N Engl J Med 363: 809–819. 20818844 10.1056/NEJMoa1002011 PMC3724529

[3] Hauschild A, Grob JJ, Demidov LV, Jouary T, Gutzmer R, et al (2012) Dabrafenib in BRAF-mutated metastatic melanoma: a multicentre, open-label, phase 3 randomised controlled trial. Lancet 380: 358–365. 22735384 10.1016/S0140-6736(12)60868-X

[4] Bollag G, Hirth P, Tsai J, Zhang J, Ibrahim PN, et al (2010) Clinical efficacy of a RAF inhibitor needs broad target blockade in BRAF-mutant melanoma. Nature 467: 596–599. 20823850 10.1038/nature09454 PMC2948082

[5] Villanueva J, Vultur A, Herlyn M (2011) Resistance to BRAF inhibitors: unraveling mechanisms and future treatment options. Cancer Res 71: 7137–7140. 22131348 10.1158/0008-5472.CAN-11-1243 PMC3588168

[6] Alcala AM, Flaherty KT (2012) BRAF inhibitors for the treatment of metastatic melanoma: clinical trials and mechanisms of resistance. Clin Cancer Res 18: 33–39. 22215904 10.1158/1078-0432.CCR-11-0997

[7] Sharma SV, Settleman J (2007) Oncogene addiction: setting the stage for molecularly targeted cancer therapy. Genes Dev 21: 3214–3231. 18079171 10.1101/gad.1609907

[8] Baselga J, Bradbury I, Eidtmann H, Di Cosimo S, de Azambuja E, et al (2012) Lapatinib with trastuzumab for HER2-positive early breast cancer (NeoALTTO): a randomised, open-label, multicentre, phase 3 trial. Lancet 379: 633–640. 22257673 10.1016/S0140-6736(11)61847-3 PMC5705192

[9] Rosell R, Carcereny E, Gervais R, Vergnenegre A, Massuti B, et al (2012) Erlotinib versus standard chemotherapy as first-line treatment for European patients with advanced EGFR mutation-positive non-small-cell lung cancer (EURTAC): a multicentre, open-label, randomised phase 3 trial. Lancet Oncology 13: 239–246. 22285168 10.1016/S1470-2045(11)70393-X

[10] Joensuu G, Joensuu T, Nupponen N, Ruutu M, Collan J, et al (2012) A phase II trial of gefitinib in patients with rising PSA following radical prostatectomy or radiotherapy. Acta Oncologica 51: 130–133. 22150168 10.3109/0284186X.2011.617387

[11] Jonsson G, Busch C, Knappskog S, Geisler J, Miletic H, et al (2010) Gene expression profiling-based identification of molecular subtypes in stage IV melanomas with different clinical outcome. Clin Cancer Res 16: 3356–3367. 20460471 10.1158/1078-0432.CCR-09-2509

[12] Busch C, Geisler J, Knappskog S, Lillehaug JR, Lonning PE (2010) Alterations in the p53 pathway and p16INK4a expression predict overall survival in metastatic melanoma patients treated with dacarbazine. J Invest Dermatol 130: 2514–2516. 20505745 10.1038/jid.2010.138

[13] Busch C, Geisler J, Lillehaug JR, Lonning PE (2010) MGMT expression levels predict disease stabilisation, progression-free and overall survival in patients with advanced melanomas treated with DTIC. Eur J Cancer 46: 2127–2133. 20541396 10.1016/j.ejca.2010.04.023

[14] Westerhuis JA, Hoefsloot HCJ, Smit S, Vis DJ, Smilde AK, et al (2008) Assessment of PLSDA cross validation. Metabolomics 4: 81–89.

[15] Kanehisa M, Goto S (2000) KEGG: Kyoto Encyclopedia of Genes and Genomes. Nucleic Acids Research 28: 27–30. 10592173 10.1093/nar/28.1.27 PMC102409

[16] Kanehisa M, Goto S, Sato Y, Furumichi M, Tanabe M (2012) KEGG for integration and interpretation of large-scale molecular data sets. Nucleic Acids Research 40: 109–114. 10.1093/nar/gkr988 PMC3245020 22080510

[17] Lai F, Jiang CC, Farrelly ML, Zhang XD, Hersey P (2012) Evidence for upregulation of Bim and the splicing factor SRp55 in melanoma cells from patients treated with selective BRAF inhibitors. Melanoma Res 22: 244–251. 22516966 10.1097/CMR.0b013e328353eff2

[18] Lai F, Jin L, Gallagher S, Mijatov B, Zhang XD, et al (2012) Histone deacetylases (HDACs) as mediators of resistance to apoptosis in melanoma and as targets for combination therapy with selective BRAF inhibitors. Adv Pharmacol 65: 27–43. 22959022 10.1016/B978-0-12-397927-8.00002-6

[19] Flaherty KT, Infante JR, Daud A, Gonzalez R, Kefford RF, et al (2012) Combined BRAF and MEK inhibition in melanoma with BRAF V600 mutations. New England Journal of Medicine 367: 1694–1703. 23020132 10.1056/NEJMoa1210093 PMC3549295

[20] Jeter JM, Cranmer LD, Hersh EM (2012) Ipilimumab pharmacotherapy in patients with metastatic melanoma. Clinical Medicine Insights Oncology 6: 275–286. 22904648 10.4137/CMO.S7245 PMC3418148

[21] Barouch-Bentov R, Sauer K (2011) Mechanisms of drug resistance in kinases. Expert Opin Investig Drugs 20: 153–208. 10.1517/13543784.2011.546344 PMC3095104 21235428

[22] Folkvord S, Flatmark K, Dueland S, de Wijn R, Groholt KK, et al (2010) Prediction of response to preoperative chemoradiotherapy in rectal cancer by multiplex kinase activity profiling. Int J Radiat Oncol Biol Phys 78: 555–562. 20675069 10.1016/j.ijrobp.2010.04.036

[23] Versele M, Talloen W, Rockx C, Geerts T, Janssen B, et al (2009) Response prediction to a multitargeted kinase inhibitor in cancer cell lines and xenograft tumors using high-content tyrosine peptide arrays with a kinetic readout. Molecular Cancer Therapeutics 8: 1846–1855. 19584230 10.1158/1535-7163.MCT-08-1029

[24] Sikkema AH, Diks SH, den Dunnen WFA, ter Elst A, Scherpen FJG, et al (2009) Kinome Profiling in Pediatric Brain Tumors as a New Approach for Target Discovery. Cancer Res 69: 5987–5995. 19567681 10.1158/0008-5472.CAN-08-3660

[25] Lindholm EM, Kristian A, Nalwoga H, Kruger K, Nygard S, et al (2012) Effect of antiangiogenic therapy on tumor growth, vasculature and kinase activity in basal- and luminal-like breast cancer xenografts. Mol Oncol 6: 418–427. 22521242 10.1016/j.molonc.2012.03.006 PMC5528356

[26] Tsai KY, Vin H, Leung M, Chitsazzadeh V, Ojeda S, et al.. (2012) Suppression of apoptosis by BRAF inhibitors through off-target inhibition of JNK signaling. 2012 ASCO Annual Meeting. Chicago, Illinois.

[27] Joseph EW, Pratilas CA, Poulikakos PI, Tadi M, Wang W, et al (2010) The RAF inhibitor PLX4032 inhibits ERK signaling and tumor cell proliferation in a V600E BRAF-selective manner. Proc Natl Acad Sci U S A 107: 14903–14908. 20668238 10.1073/pnas.1008990107 PMC2930420

[28] Halaban R, Zhang W, Bacchiocchi A, Cheng E, Parisi F, et al (2010) PLX4032, a selective BRAF(V600E) kinase inhibitor, activates the ERK pathway and enhances cell migration and proliferation of BRAF melanoma cells. Pigment Cell Melanoma Res 23: 190–200. 20149136 10.1111/j.1755-148X.2010.00685.x PMC2848976

[29] Poulikakos PI, Zhang C, Bollag G, Shokat KM, Rosen N (2010) RAF inhibitors transactivate RAF dimers and ERK signalling in cells with wild-type BRAF. Nature 464: 427–430. 20179705 10.1038/nature08902 PMC3178447

[30] Weber CK, Slupsky JR, Kalmes HA, Rapp UR (2001) Active Ras induces heterodimerization of cRaf and BRaf. Cancer Res 61: 3595–3598. 11325826

[31] Dumaz N, Hayward R, Martin J, Ogilvie L, Hedley D, et al (2006) In melanoma, RAS mutations are accompanied by switching signaling from BRAF to CRAF and disrupted cyclic AMP signaling. Cancer Res 66: 9483–9491. 17018604 10.1158/0008-5472.CAN-05-4227

[32] Sensi M, Nicolini G, Petti C, Bersani I, Lozupone F, et al (2006) Mutually exclusive NRASQ61R and BRAFV600E mutations at the single-cell level in the same human melanoma. Oncogene 25: 3357–3364. 16462768 10.1038/sj.onc.1209379

[33] Dahlman KB, Xia J, Hutchinson K, Ng C, Hucks D, et al (2012) BRAFL597 mutations in melanoma are associated with sensitivity to MEK inhibitors. Cancer discovery 2: 791–797. 22798288 10.1158/2159-8290.CD-12-0097 PMC3449158

[34] Tap WD, Gong KW, Dering J, Tseng Y, Ginther C, et al (2010) Pharmacodynamic characterization of the efficacy signals due to selective BRAF inhibition with PLX4032 in malignant melanoma. Neoplasia 12: 637–649. 20689758 10.1593/neo.10414 PMC2915408

[35] Sondergaard JN, Nazarian R, Wang Q, Guo DL, Hsueh T, et al.. (2010) Differential sensitivity of melanoma cell lines with BRAF(V600E) mutation to the specific Raf inhibitor PLX4032. Journal of Translational Medicine 8. 10.1186/1479-5876-8-39 PMC2876068 20406486

[36] Poulikakos PI, Persaud Y, Janakiraman M, Kong X, Ng C, et al (2011) RAF inhibitor resistance is mediated by dimerization of aberrantly spliced BRAF(V600E). Nature 480: 387–390. 22113612 10.1038/nature10662 PMC3266695

[37] Paraiso KH, Xiang Y, Rebecca VW, Abel EV, Chen YA, et al (2011) PTEN loss confers BRAF inhibitor resistance to melanoma cells through the suppression of BIM expression. Cancer Res 71: 2750–2760. 21317224 10.1158/0008-5472.CAN-10-2954 PMC3070772

[38] Nazarian R, Shi H, Wang Q, Kong X, Koya RC, et al (2010) Melanomas acquire resistance to B-RAF(V600E) inhibition by RTK or N-RAS upregulation. Nature 468: 973–977. 21107323 10.1038/nature09626 PMC3143360

[39] Prahallad A, Sun C, Huang S, Di Nicolantonio F, Salazar R, et al (2012) Unresponsiveness of colon cancer to BRAF(V600E) inhibition through feedback activation of EGFR. Nature 483: 100–103. 22281684 10.1038/nature10868

[40] Villanueva J, Vultur A, Lee JT, Somasundaram R, Fukunaga-Kalabis M, et al (2010) Acquired resistance to BRAF inhibitors mediated by a RAF kinase switch in melanoma can be overcome by cotargeting MEK and IGF-1R/PI3K. Cancer Cell 18: 683–695. 21156289 10.1016/j.ccr.2010.11.023 PMC3026446

